# Genome-Wide Identification and Expression Analyses of the Chitinases under Cold and Osmotic Stress in *Ammopiptanthus nanus*

**DOI:** 10.3390/genes10060472

**Published:** 2019-06-21

**Authors:** Shilin Cao, Ying Wang, Zhiqiang Li, Wei Shi, Fei Gao, Yijun Zhou, Genfa Zhang, Jinchao Feng

**Affiliations:** 1 College of Life and Environmental Sciences, Minzu University of China, Beijing 100081, China; csl_muc@163.com (S.C.); wangyinggggg@126.com (Y.W.); ynlflzq@163.com (Z.L.) zhouyijun@muc.edu.cn (Y.Z.); jchfeng@263.net (J.F.); 2 Key Laboratory of Biogeography and Bioresource in Arid Land, Institute of Ecology and Geography in Xinjiang, The Chinese Academy of Sciences, Urumqi 830011, Xinjiang, China; water5116@163.com; 3 College of Life Sciences, Beijing Normal University, Beijing 100875, China; gfzh@bnu.edu.cn

**Keywords:** chitinase, *Ammopiptanthus nanus*, osmotic stress, low temperature, gene family

## Abstract

Chitinase is a kind of hydrolase with chitin as a substrate and is proposed to play an essential role in plant defense system by functioning against fungal pathogens through degrading chitin. Recent studies indicated chitinase is also involved in abiotic stress response in plants, helping plants to survive in stressful environments. *A. nanus*, a rare evergreen broad-leaved shrub distrusted in deserts in Central Asia, exhibits a high level of tolerance to drought and low temperature stresses. To identify the chitinase gene involved in drought and low temperature responses in *A. nanus*, we performed genome-wide identification, classification, sequence alignment, and spatio-temporal gene expression analysis of the chitinases in *A. nanus* under osmotic and low temperature stress. A total of 32 chitinase genes belonging to glycosyl hydrolase 18 (GH18) and GH19 families were identified from *A. nanus*. Class III chitinases appear to be amplified quantitatively in *A. nanus*, and their genes carry less introns, indicating their involvement in stress response in *A. nanus*. The expression level of the majority of chitinases varied in leaves, stems, and roots, and regulated under environmental stress. Some chitinases, such as *EVM0022783*, *EVM0020238*, and *EVM0003645*, are strongly induced by low temperature and osmotic stress, and the MYC/ICE1 (inducer of CBF expression 1) binding sites in promoter regions may mediate the induction of these chitinases under stress. These chitinases might play key roles in the tolerance to these abiotic stress in *A. nanus* and have potential for biotechnological applications. This study provided important data for understanding the biological functions of chitinases in *A. nanus*.

## 1. Introduction

Chitinase is a type of hydrolase with chitin as a substrate, and chitin is a glycopolymer of β-1,4-linked N-acetyl-D-glucosamine. Chitin was mostly found in animal exoskeletons, the inner wall of the digestive tract, and the fungal cell wall, but not in plants [1]. Fungi are the most important plant pathogens which cause significant crop yield losses every year. During the long-term evolution process, plants developed an innate immune system and trigger defense response against invading pathogens upon the perception of various immunogenic microbial signatures, called microbe-associated molecular patterns (MAMPs), or pathogen-associated molecular patterns (PAMPs) [2,3]. Plant chitinase catalyzes the hydrolysis of β-1, 4 glycosidic bonds in chitin in fungal cell walls and releases chitin oligosaccharides (CTOS) during fungal infection [4,5]. As plants do not contain chitin, CTOS is recognized as a non-self component and a kind of PAMP, and activates the host immune responses of plants [6,7]. Thus, as one of the functional families of pathogenesis-related proteins (PRs), chitinases play key roles in the plant defense system by protecting plants from chitin-containing pathogens such as fungi [8], and are considered important target genes for crop improvement [1]. In addition, although the endogenous substrate of plant chitinase is not determined, several chitinases are suggested to cleave arabinogalactan proteins (AGPs) [9] and N-acetylglucosamine-containing glycoproteins in the plant cell walls [10], and released oligosaccharides that might act as signal molecules triggering a defense response in plants.

Based on the similarity of amino acid sequences, plant chitinases can be divided into five classes, namely class I–V, wherein class III and class V belong to the GH (glycosyl hydrolase) 18 family, and class I, class II and class IV belong to the GH19 family. GH18 is present in plants, animals, fungi and viruses, while GH19 is only present in plants [11]. Class I has a highly-conserved N-terminal cysteine-rich region and a chitin-binding region, usually with a C-terminal extension. Class II lacks an N-terminal cysteine-rich region and a chitin-binding region, but its catalytic region is highly similar to the amino acid sequence of class I chitinase. Class IV chitinases have a chitin-binding region and a catalytic domain. Class III lacks a chitin-binding region and has little sequence identity to GH19 chitinase. Class V does not have a chitin-binding region, which is more similar to fungal and bacterial chitinase than other plant chitinases [1].

More and more evidences indicated that in addition to playing an important role in disease defense such as anti-fungi, plant chitinase has other biological functions, including participation in plant development [12,13], symbiotic interactions between eukaryotes and microbes [14], pollination, senescence, seed germination, somatic embryogenesis [1], hormone response [15], and response to drought and low-temperature stress [16,17]. Some chitinases were involved in the low-temperature tolerance of plants and helped plants to survive in low temperature environments, and those chitinases exhibited antifreeze activity in hardy plant species including *Bromus inermis*, *Picea pungens*, and *Chimonanthus praecox* [18,19,20]. The expression level of a chitinase was induced in young green leaves by salicylic acid or methyl jasmonate treatment in *Capsicum annuum* [21]. Ectopic expression of a sugarcane chitinase promoted the growth rate of *E. coli* under NaCl, CuCl_2_, CdCl_2_, and ZnSO_4_ treatment [22]. A class I chitinase induced by methyl jasmonate and low temperature might be regulated through the CBF (C-repeat binding factor)/ERF (ethylene response factor)-dependent cold stress signaling pathway in *Hippophae rhamnoides* [23]. 

*Ammopiptanthus nanus* belongs to the Ammopiptanthus, Leguminosae, which is a tertiary relict plant in Central Asia. It is a rare evergreen broad-leaved shrub species, mainly distributed in the barren hills at the junction of Kunlun Mountain and Pamir in the southern Kashgar region. *A. nanus* can tolerate extremely high and low temperature and severe drought stress, and in the Gobi area, the typical habitat of *A. nanus*, the annual temperature fluctuation range is from −30 °C to 40 °C. Considering the roles of chitinases in environmental stress, we speculate that some number of the chitinase family might contribute to the high level of abiotic stress tolerance in *A. nanus.* The identification of chitinase family and the investigation of their expression pattern under stress condition can greatly promote the understanding of the biological functions of chitinase, as revealed by the studies on chitinase family in *Arabidopsis thaliana* [24], *Eucalyptus grandis* [25], *Populus trichocarpa* [26], *Solanum lycopersicum* [27], *Hevea brasiliensis* [28], *Brassica rapa* [6], *Brassica juncea* [29], *Camelina sativa* [29], sugarcane [30], and four cotton species [31]. 

In recent years, high-throughput sequencing technology has provided a great deal of nucleic acid data for *A. nanus* [32], especially recently, the whole genome sequencing was completed using single-molecule sequencing [33], which makes it possible to identify chitinase family at the genome level. In the present study, the genome-wide identification of chitinase in *A. nanus* was conducted and expression profiling of the chitinase family under cold and osmotic stress was performed using quantitative real-time PCR (qRT-PCR). Our study will provide important data for the understanding of the biological roles of chitinases in *A. nanus*.

## 2. Materials and Methods 

### 2.1. Identification of Putative Chitinase Genes

To identify the chitinase genes in A. nanus, the genome sequence and annotation data were obtained from the A. nanus genome project [33]. The Hidden Markov Model (HMM) seed profile of the Glyco_hydro_18 (PF00704) and Glyco_hydro_19 (PF00182) was downloaded from the Pfam database [34], and chitinase genes in A. nanus were identified using HMMER3 (v. 3.0) software [35]. Then, all predicted chitinase genes were manually checked to confirm the presence of the conserved domains of Glyco_hydro_18 or Glyco_hydro_19.

### 2.2. Multiple Alignment and Phylogenetic Analysis

Sequence alignment of the full length of the amino acid sequences of GH18 and GH19 was performed using clustalW [36]. Considering that these two families have no similar sequences, and a single phylogenetic tree cannot satisfy the phylogenetic analysis of the two families, we performed phylogenetic analysis on the GH18 and GH19 families separately. The phylogenetic tree is constructed using MEGA-X [37]. The evolutionary history was inferred by using the Maximum Likelihood method based on the Poisson correction model [38]. Initial trees for the heuristic search were obtained automatically by applying Neighbor-Join and BioNJ algorithms to a matrix of pairwise distances estimated using a Jones-Taylor-Thornton (JTT) model, and then selecting the topology with superior log likelihood value. The tree is drawn to scale, with branch lengths measured in the number of substitutions per site. The analysis involved 34 amino acid sequences. All positions containing gaps and missing data were eliminated. There were 48 positions in the final dataset.

### 2.3. Identification of Conserved Domains

Dialign-Pfam [39] and MEME suite 5.0.1 (Multiple Em for Motif Elicitation) [40] were used to identify conserved domains with the default parameters. BioEdit [41] was used for sequence alignment and editing. According to the homology of amino acid sequences, the multiple sequence alignment was conducted separately in three groups: group 1 comprised of chitinases of class I, class II, and class IV; group 2 contained class III chitinases; group 3 included several class V chitinases.

### 2.4. Visualization of Chitinase Genes on Chromosomes

The chromosomal locations of all identified chitinase genes were visualized by using Mapchart 2.3 [42] based on the gene annotation information available at the B. rapa genome database.

### 2.5. Prediction of Cis-Acting Elements in Promoter Regions of Chitinase Genes

The online tool PLACE [43] was used to predict the cis-acting elements in promoter region (1000 bp upstream of the start codon) of chitinases in A. nanus.

### 2.6. Signal Peptide Prediction 

The online tool SignalP 4.1 server [44] was used to judge whether a chitinase contains a signal peptide based on the C, Y, and S values.

### 2.7. Plant Materials and Stress Treatments

The seeds of *A. nanus* were collected from Wuqia county, Xinjiang autonomous district, China. The seeds were surface sterilized using 70% (v/v) ethanol for 1 min, followed by bleaching (10%) for 6 min, and then were planted in a 30 cm diameter pot containing a 3:1 (v/v) mixture of vermiculite and perlite. Seedlings were grown in a growth chamber under 120 µmol m^−2^ s^−1^ photosynthetic photon flux density, with a photoperiod of 16h light and 8h dark cycle, at approximately 25 °C and 35% relative humidity. The seedlings were watered every 4 days with half-length Hoagland solution. Eight weeks after germination, seedlings with uniform growth were selected and were subjected to low temperature and osmotic stress treatment.

For osmotic stress treatments, the seedlings were randomly divided into five groups. The four osmotic stress-treated groups were irrigated with 20% PEG-6000 for 3 h, 6 h, 12 h, or 24 h, whereas the unstressed group was used as the control. For low-temperature stress treatments, the seedlings were randomly divided into five groups. The four low temperature stress-treated groups were moved to a growth chamber at 4 °C for 3 h, 6 h, 12 h, or 24 h, and the unstressed group was used as the control. All leaf samples of the control and treated groups were collected, snap-frozen in nitrogen, and stored at −80 °C until further RNA extraction.

### 2.8. qRT-PCR Analysis of the Chitinase Genes in A. nanus

Total RNA samples were extracted using the Trizol reagent following the manufacturer’s directions (Invitrogen, CA, USA). The qRT-PCR analyses were conducted according to a previously described method [45]. Three independent biological replicates for each group and three technical replicates of each biological replicate were analyzed using qRT-PCR. The expression levels of A. nanus were normalized against an internal reference gene, 18S rRNA. The relative gene expression was calculated using the 2^−ΔΔCt^ method [46]. Standard deviations were calculated from three biological replicates. Primer3 v. 4.0 [47] was used to design primers for qRT-PCR of chitinase genes (Table S1). Since one of the chitinase genes (EVM0003584) failed to be amplified, the experiment contained only the qRT-PCR results of 31 chitinase genes.

### 2.9. Statistics

Student *t* tests were used to test for significant differences between the control and treatment group. *p* < 0.05 was considered significant. Chitinase genes were considered to be differentially expressed if they show a fold-change of at least two and also satisfy *p* < 0.05 compared to the control group.

## 3. Results

### 3.1. Genome-wide Identification of chitinase Genes in A. nanus

**Figure 1:**
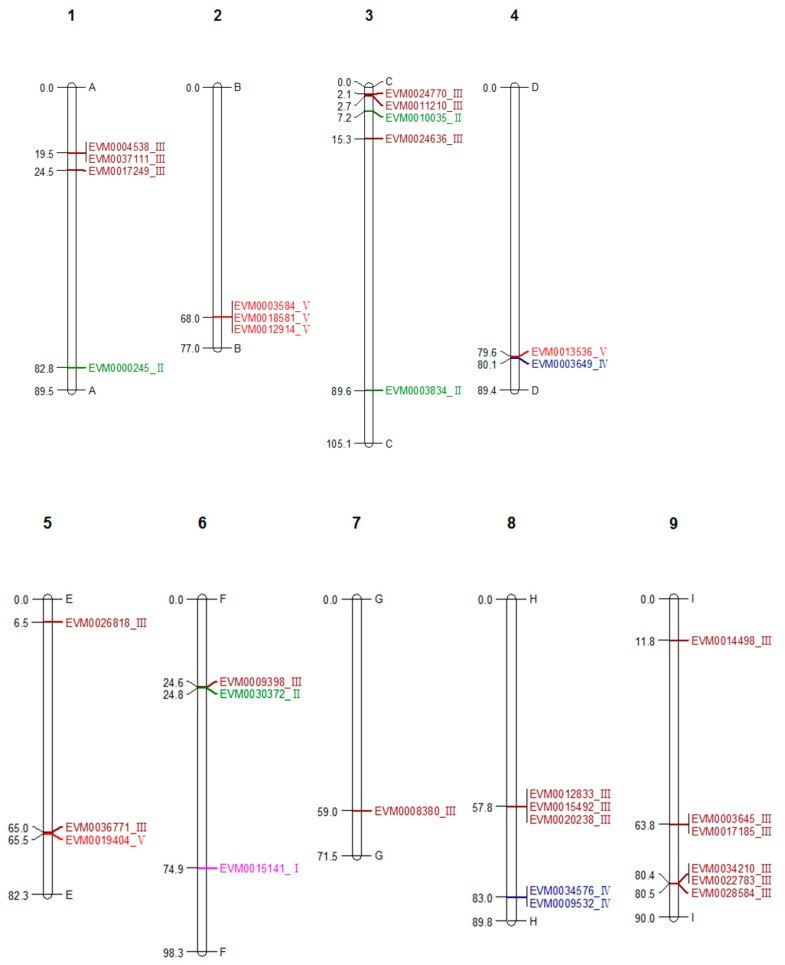
Physical mapping of all identified *A. nanus* chitinases from the two glycosyl hydrolase families: GH18 (red /brown) and GH19 (blue /pink/ green). The major classes are represented as follows: pink = class I, green = class II, brown = class III, blue = class IV and red = class V. Scale bar represents Mb. The Roman number following the gene ID indicates its class.

A total of 32 chitinase genes were identified from the genome sequence of *A. nanus*, and out of these chitinases, 24 were classified into the GH18 subfamily, and 8 into the GH19 subfamily (Table 1 and Figure 1). According to the classification criteria of plant chitinases described previously [24,48], there is a single class I, 4 class II, 19 class III, 3 class IV, and 5 class V chitinases in *A. nanus*.

The 32 predicted chitinases range in length from 119 to 1027 amino acid residues, with 27 chitinase genes falling in length range from 245 to 441 amino acid residues. The top three longest chitinases were EVM0018581, EVM0003645, and EVM0017185, with amino acid sequences longer than 700 amino acids. Considering that the length of these three chitinases are significantly larger than that of other chitinases, it is speculated that they may contain other domains. Using the Conserved Domain Search Service (CD Search tool) (https://www.ncbi.nlm.nih.gov/Structure/cdd/wrpsb.cgi) in the NCBI website [49], the three longest chitinases were aligned to the conserved domains in the database and the result showed that there is a Protein tyrosine kinase (Pkinase Tyr, PF07714) domain at the carboxyl end of these three chitinases. The pI values ranged from 4.22 (EVM0034576) to 9.12 (EVM0013536), with 13 chitinases showing a pI value > 7. The only class I chitinase is alkaline, all class IV chitinases are acidic, most class III chitinases are acidic, and the majority of class V chitinases are alkaline. Not all chitinases contain signal peptides, and two chitinases in class IV, EVM0034576 and EVM0009532, and two chitinases in class III, EVM0017249 and EVM0036771, do not contain predicted signal peptide sequences. A total of 28 chitinases have signal peptides at the amino end, indicating that most of the *A. nanus* chitinases are secreted to apoplast. Class I and class IV chitinase, excluding EVM0009532, contain a cysteine-rich N-terminal chitin-binding domain (CBD). The catalytic domains were missed in class I, class V chitinases, and several class III chitinases.

### 3.2. Multiple Alignment and Phylogenetic Analysis

**Figure 2:**
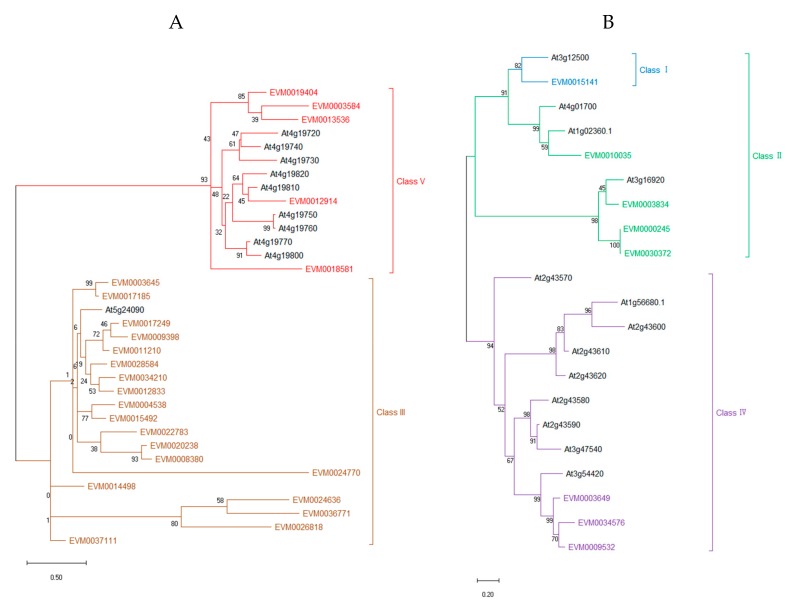
Phylogenetic trees of *A. nanus* chitinase genes from two glycosyl hydrolase families: GH18 (**A**), including class III (green) and V (blue) chitinase genes, and GH19 (**B**), including class IV (violet), class II (green), and class I (blue) chitinase genes. *EVM0015141* was the only class I chitinase. The tree with the highest log-likelihood (-2667.05) is shown. The percentage of trees in which the associated taxa clustered together is shown next to the branches.

Considering that the GH18 and GH19 share little sequence similarity, the GH18 and GH19 amino acid sequences were aligned and analyzed phylogenetically separately. As shown in Figure 2, the phylogenetic trees of chitinase genes from *A. nanus* and *A. thaliana* are shown in two graphs, GH18 (Figure 2A) and GH19 (Figure 2B). The chitinase genes within the same GH families show a high level of similarity. There are two large clades in the phylogenetic trees of both GH18 and GH19 families. All class V and class III *A. nanus* chitinases, together with the chitinases of the same classes from *A. thaliana*, formed the two large clades in the phylogenetic trees of GH18. *A. nanus* chitinases belonged to class I, II, and IV, as well as the corresponding chitinases from *A. thaliana*, constituted the two large clades in the phylogenetic tree of GH19. The only one class I chitinase in *A. nanus*, clustering with the only one class I chitinase from *A. thaliana*, was present in the clade which mainly consisted of class II chitinases [50].

As shown in Figure 2, the total number of chitinase genes in *A. nanus* was eight more than that of *A. thaliana*. The number of both class I and class II chitinases in *A. nanus* and *A. thaliana* was the same, while the numbers of class IV and class V chitinases in *A. nanus* were smaller than those of *A. thaliana*. The number of class IV chitinases of *A. nanus* and *A. thaliana* are three and nine, and the number of class IV chitinases of *A. nanus* and *A. thaliana* are five and nine, respectively. The number of class III chitinases in *A. nanus* and *A. thaliana* is 19 and 1, respectively. Class I and class II chitinases are not clearly divided and are highly proximate in the phylogenetic tree (Figure 2), which supports the hypothesis that class II chitinases were derived from the perspective of class I [51].

Since some class III chitinases were clustered in *A. nanus* chromosome 1, 3, 8, and 9 (Figure 1), we wonder if the number of class III was increased compared with other plant species. Thus, we compared the number of each chitinase class between *A. nanus* and some other plant species (Table 2), and found that, compared with *A. thaliana*, *B. rapa*, *P. trichocarpa*, *E. grandis*, *G. raimondii*, and *H. brasiliensis*, there were relatively more class III chitinases in *A. nanus*. Class III chitinases appear to be amplified in *A. nanus*.

### 3.3. Conserved Domains

**Figure 3:**
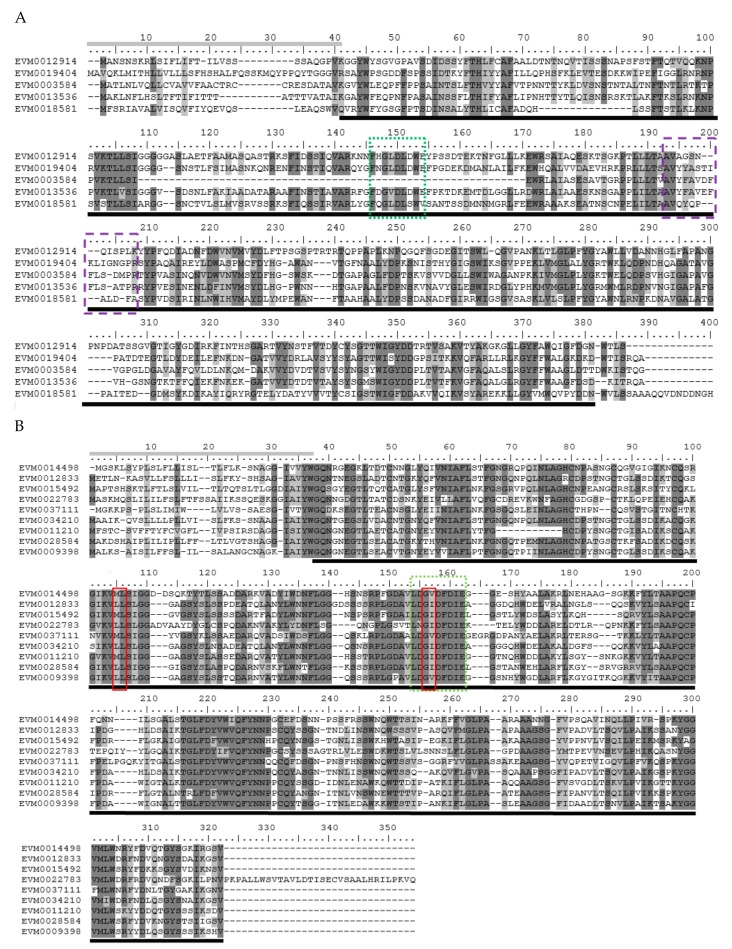
Multiple alignments of *A. nanus* chitinases in GH18 family class V (**A**) and class III (**B**). Sequence alignment was performed using ClustalW and edited using BioEdit. Shaded amino acid sequences are 70–100% homologous. Underlined sequences represent domain homology for GH18. Gray line indicates signal sequence. The amino acids in the red box represent residues essential for catalytic activity. Purple box (dash line) = CRYSTALLYN_BETAGAMMA signature PS00225 ([LIVMFYWA]-{DEHRKSTP}-[FY]-[DEQHKY]-x(3)-[FY]-x-G-x(4)-[LIVMFC-ST]) and green box (dotted line) = Chitinase_18 signature PS01095 ([LIVMFY]-[DN]-G-[LIVMF]- [DN]-[LIVMF]-[DN]-x-E).

**Figure 4:**
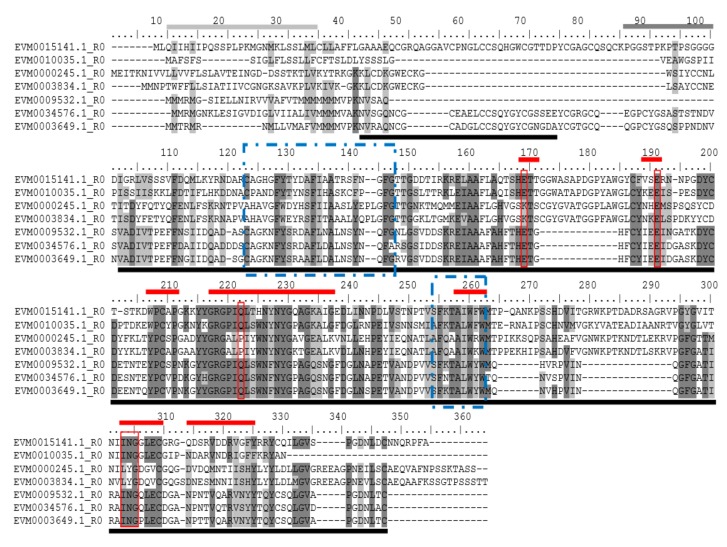
Multiple alignment of *A. nanus* chitinases in GH19 family. Sequence alignment was performed using ClustalW and edited using BioEdit. Underlined indicates the homologous domains that constitute the chitin-binding domain (amino acid residues 42-74) and GH19 (amino acid residues 102-347). The red line above the sequence indicates the active site defined by the amino acid residue. The light gray line above the sequence indicates the signal sequence and the dark gray line (amino acid residues 86–100) indicates the hinge region rich in proline/glycine. Amino acid residues in the red box are critical for the catalytic activity or enzymatic function of class I. *EVM0010035*, *EVM0000245*, and *EVM0003834* do not have chitin-binding domains, indicating that they belong to class II. For class I and class II. Blue box 1 (dot-dash line) = chitinase 19_1 signature PS00773 (Cx(4,5)-FY-[ST]-x(3)-[FY]-[LIVMF]-xAx(3)-[YF ]-x(2)-F--[GSA]); blue box 2 (dot-dash line) = Chitinase 19_2 signature PS00774 ([LIVM]-[GSA]-Fx-[STAG](2)-[ LIVMFY]-W-[FY]-W-[LIVM]).

To locate the conserved domains in chitinases of *A. nanus*, we conducted multiple sequence alignment and motif-based sequence analysis for chitinases in class V (Figure 3A); class III (Figure 3B); and class I, II, and IV (Figure 4). Firstly, the amino acid sequence homologous to the active sites of chitinases was found in all the chitinases used for analysis ( Figure 3; Figure 4, boxed in Red). It is noteworthy that, compared with class I and class II chitinases, there are three distinct deletions (amino acid residues 172–184, 265–271, and 279–293) in the three class IV chitinases, i.e., EVM0003649, EVM0034576, and EVM0009532 (Figure 4). These three deletions are indeed characteristic of class IV chitinases [1], which make class IV chitinases and type 1 clustered into two clades in the phylogenetic tree of GH19 (Figure 2B).

The class I and class IV chitinases in *A. nanus* contain the chitin-binding domain and the GH19 catalytic domain. Unlike the chitinase class II in eucalyptus, which has no chitin-binding domain or a catalytic domain [25], class II in *A. nanus* chitinase has a GH19 catalytic domain but no chitin-binding domain. Class III and class V have the GH18 catalytic domains, and class V also harbors the chitin-binding domain; however, no chitin-binding domain was found in class III chitinases.

### 3.4. Intron-Exon Architecture

**Figure 5:**
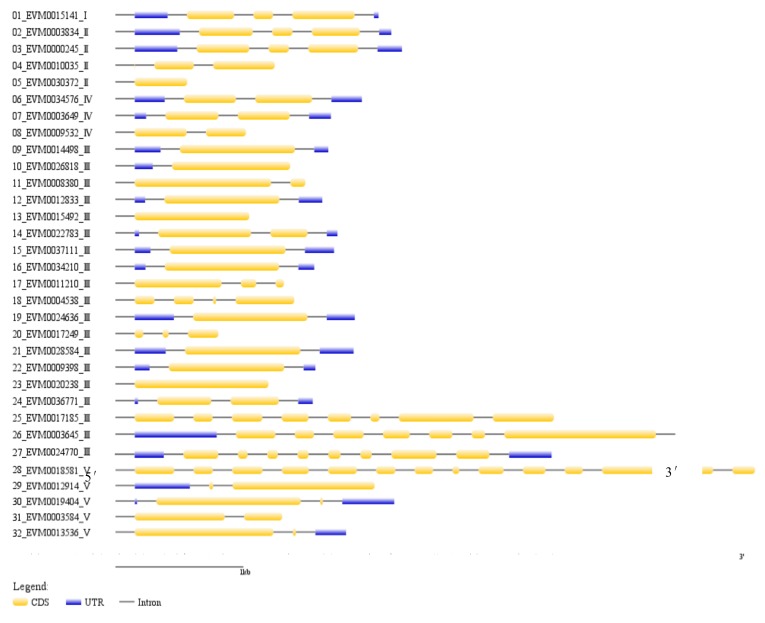
The intron-exon structure of chitinase gene family in *A. nanus*. The yellow horizontal bar indicates coding sequences (CDS), blue horizontal bar indicates untranslated regions (UTR), and black line indicates intron. The Roman number following the gene ID indicates its class.

Among the 32 chitinases in *A. nanus*, 28 chitinases have less than five introns, including 3 chitinases with no introns, 4 with one, 12 with two, 6 with three, and 3 with four introns (Figure 5). Four chitinases with long amino acid sequences contain more than seven introns, including the three chitinases harboring the PKc domain, i.e., EVM0017185, EVM0003645, and EVM0018581. 

### 3.5. Prediction of the Cis-Acting Elements in Promoter Region of the Chitinase in A. nanus

**Figure 6:**
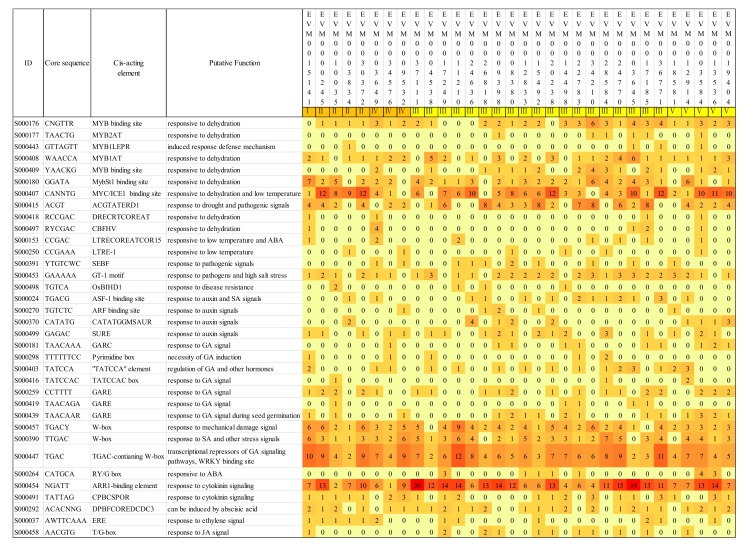
Prediction of the cis-acting elements in the promoter region of the chitinases in *A. nanus.* The color of the square indicates the number of cis-acting elements in the promoter region. The redder the square color, the more cis-acting elements there are.

To determine the cis-acting elements which may be associated with the spatial and temporal expression patterns of chitinases, we predicted the cis-acting elements from the 1000 bp upstream sequences of each chitinase in genome. A number of cis-acting elements involved in the response to drought, low temperature, and phytohormones were predicted (Figure 6). Among these cis-acting elements, MYC/ICE1 (inducer of CBF expression 1) binding site [52], ACGTATERD1 [53], MYB binding site [52], and the other seven elements are reported to participate in the drought response; MYC/ICE1 binding site, LTRECOREATCOR15 [54], and LTRE-1 are associated to a low-temperature response; 4 cis-acting elements participated in the pathogen response; and 19 cis-acting elements (SURE, GARE, W-box [55], T/G-box, etc.) are involved in the response to phytohormones such as auxin, gibberellin, cytokinin, and salicylic acid. 

The occurrence frequency of different cis-acting elements varies greatly. The most frequently occurring cis-acting elements in all promoters of the chitinase family are MYC/ICE1-binding site, TGAC-containing W-box, and ARR1-binding element [56], which are involved in the response to drought and low temperature, GA, and cytokinin, respectively (Figure 6). 

### 3.6. Expression Levels of A. nanus Chitinases in Leaf, Stem, and Root 

**Figure 7:**
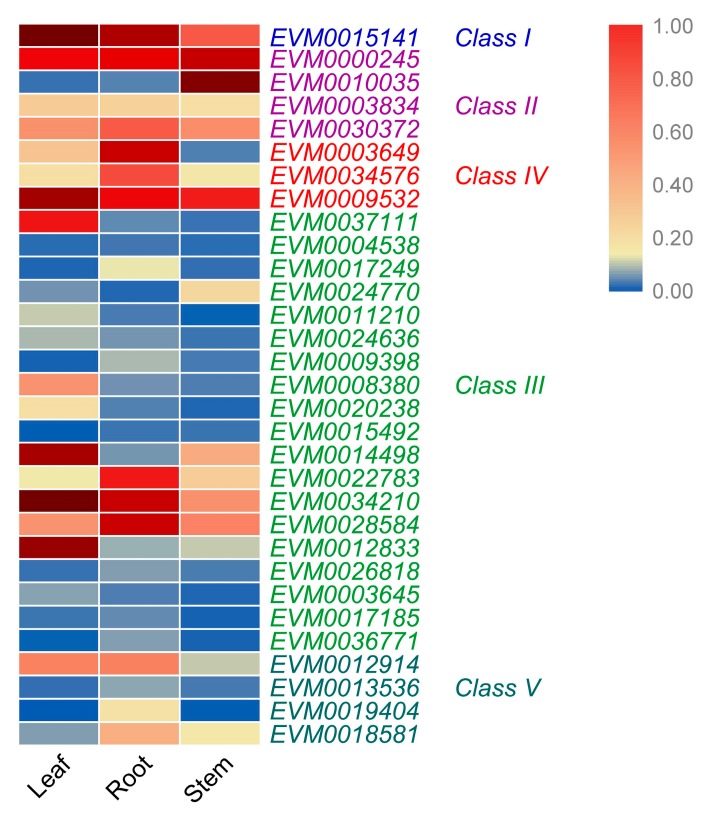
Expression levels of *A. nanus* chitinases in leaves, stems, and roots. *A. nanus* elF1 was used as the internal control.

qRT-PCR analyses were conducted to analyze the tissue-specific expression pattern of chitinase in *A. nanus*; the results showed that the expression levels of chitinase genes in roots, stems and leaves were obviously different (Figure 7 and Figure S1). Ten chitinase genes exhibited a high expression level in leaves (Figure S1A); 11 chitinases, including *EVM0026818*, *EVM0034576*, and *EVM0003649*, expressed dominantly in roots (Figure S1B); and the other three chitinases, *EVM0010035, EVM0024770*, and *EVM0000245* expressed highly in stems (Figure S1C). The expression of the remaining seven chitinases did not show obvious tissue specificity (Figure S1D). In general, most of the chitinases in *A. nanus* exhibited a tissue-specific expression pattern.

### 3.7. Expression Pattern of A. nanus Chitinases under Low Temperature Stress

**Figure 8:**
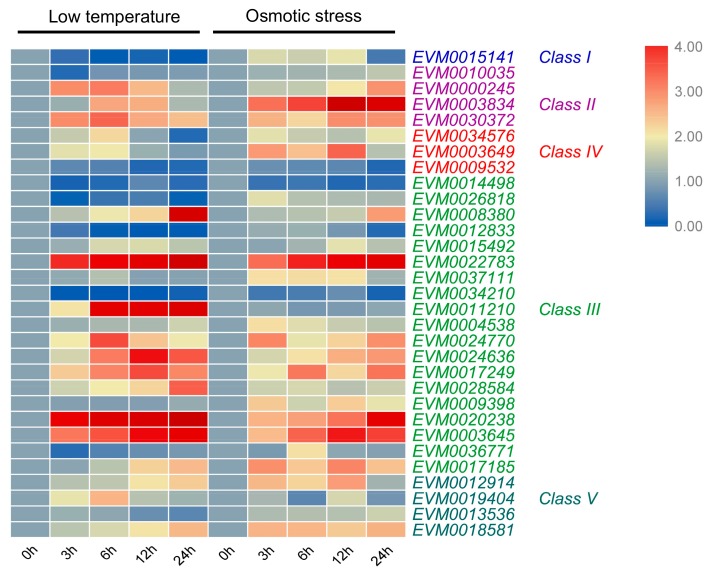
Expression pattern of *A. nanus* chitinases under low temperature and osmotic stress. *A. nanus* elF1 was used as the internal control.

To identify chitinase involved in low temperature response, qRT-PCR was conducted to investigate the expression pattern of chitinase family in *A. nanus* under low-temperature treatment (Figure 8 and Figure S2). The majority of chitinases (20/31) were up-regulated under low temperature stress in *A. nanus* leaves. According to their expression pattern, the cold-induced chitinase were further categorized into two groups. The expression pattern of the first group shows a linear upward curve, and this group includes 10 chitinase genes (Figure S2A). The expression pattern of another group exhibited an inverted U-shaped curve, and this group is composed of 10 chitinase genes including *EVM0034576*, *EVM0003649*, and *EVM0000245* (Figure S2B).

The expression levels of eight chitinase, such as *EVM0014498*, *EVM0010035*, and *EVM0026818*, were down-regulated under low temperature treatment (Figure S2C), and the expression of *EVM0037111, EVM0013536,* and *EVM0009398* did not show significant change (up or down-regulated by >2-fold) under low-temperature stress (Figure S2D). 

### 3.8. Expression Pattern of A. nanus Chitinases under Osmotic Stress

To identify chitinase involved in drought stress response, qRT-PCR was conducted to investigate the expression pattern of chitinase family in *A. nanus* under osmotic stress (Figure 8 and Figure S3). Most chitinases (26/31) were up-regulated under osmotic stress in *A. nanus* leaves. Based on their expression patterns, the osmotic stress-induced chitinase were further categorized into two groups. The expression pattern of the first group shows a linear increase curve, and this group is composed of seven chitinase genes (Figure S3A). The expression level of the second group was up-regulated by > two-fold during at least one time-point under osmotic stress, but cannot be classified into the first group, and this group includes 19 chitinase genes (Figure S3B).

The expression levels of four chitinase, i.e., *EVM0014498*, *EVM0012833*, *EVM0034210*, and *EVM0009532*, were down-regulated under osmotic stress (Figure S3C), and the expression of *EVM0011210,* did not show significant change (up or down-regulated by > two-fold) under osmotic stress (Figure S3D). Although many chitinases were responsive to both low temperature and osmotic stress, seven chitinases, namely, *EVM0010035*, *EVM0026818*, *EVM0015141*, *EVM0036771*, *EVM0037111, EVM0013536,* and *EVM0009398* were induced only by osmotic stress. 

## 4. Discussion

*A. nanu* can survive in arid regions with extremely high levels of low temperature and drought stress in Central Asia, and a number of studies were conducted to identify the stress-related genes [57,58]. Here, based on the high-quality genome completed recently [33], we performed a genome-wide identification of putative chitinase gene family in *A. nanus*. A total of 32 chitinase genes were found in *A. nanus*, including 24 chitinases belong to the GH18 subfamily, and eight within the GH19 subfamily. The total number of chitinase is comparable to that of *B. rapa*, *A. thaliana*, and *P. trichocarpa*, but significantly lower than that of *E. grandis*, in which genome the chitinase family was proposed to be amplified [25]. 

Although the 32 chitinase genes are distributed in the nine chromosomes of the *A. nanus* (Figure 1), the distribution of chitinases on the chromosome is uneven: more chitinases are located in chromosome 3, 8, and 9, and only one chitinase is present in chromosome 7. Some chitinases were even clustered on some individual chromosomes. For example, three class III chitinases and two class III chitinases were clustered on chromosome 8 and 1, respectively. Most of the clustered genes belong to the same family. For instance, on chromosome 1, both *EVM0012914* and *EVM0003584* belong to class V, and the chitinase clustered on chromosome 8, *EVM0012833*, *EVM0015492*, and *EVM0020238*, belong to class III. The distribution pattern of chitinases in *A. nanus* in the chromosome is similar to those of other species such as *S. lycopersicum* [27] and *E. grandis* [25].

By comparing with *A. thaliana*, *B. rapa*, *P. trichocarpa*, *E. grandis*, and other plant species, we found that the number of class III chitinases in *A. nanus* were significantly higher than that of other plant species. Although statistics of the chitinase family in more plant species is still needed to draw a reliable conclusion, our data support the hypothesis that class III was amplified in *A. nanus*. Unlike class I, II, and IV plant chitinases, class III chitinases exhibit higher sequence similarity with yeast chitinases, and some class III chitinases possess a lysozyme activity which is not found in other classes of chitinases [59], which indicate that plant class III chitinases might have different biological roles than other classes of chitinases [51]. For example, a class III chitinase with β-1,3-glucanase, β-1,4 glucanase, and lysozyme activity, inhibited the growth of non-symbiotic bacteria, but did not affect the growth of the symbiotic bacteria *Frankia*, thus playing a key role in the symbiotic process in *Casuarina glauca* nodules [60]. The only class III chitinase in Arabidopsis, LYS1 (At5g24090), is implicated in immunity to bacterial infection by breaking down peptidoglycans and releasing PGN-derived PAMP [61]. A soybean class III chitinase was shown to be involved in defense, development and dormancy events in seeds [62], and ectopic expression of ScChi, a class III chitinase of sugarcane, enhances the tolerance to both biotic and abiotic stresses in tobacco [17]. Anyway, more research is needed to clarify the differences in biological functions between class III chitinase and other types of chitinase.

In the long evolutionary process, some eukaryotic lineages have undergone obvious intron loss [63]. In general, intron density is inversely proportional to the rapid regulation of stress response, and stress-related genes contain fewer introns [64]. Given that the average number of introns in the *A. thaliana* protein coding gene is 4.38 [65], the average number of introns in the chitinase family in *A. nanus* is relatively small, which is 2.93, especially, apart from the four chitinases with a length more than 440 AA; the average intron number of the remaining 28 chitinases is only 2.07. Excluding the three chitinases with long amino acid sequences (EVM0017185, EVM0003645, and EVM0024770), the average number of introns in the remaining class III chitinases in *A. nanus* is as low as 1.81, in which supporting class III chitinases might play important roles in rapid response to environmental stresses in *A. nanus*. Furthermore, except for *EVM0003584* that was not analyzed using qRT-PCR, five of the remaining six chitinase genes without introns or with only one intron were up-regulated by low temperature and osmotic stress (Figure 8), which is consistent with the hypothesis that intron-deficient genes are rapidly regulated during stress [64].

Many chitinase genes within the same class or the same subclass of the phylogenetic tree showed similar expression patterns. For example, all three chitinase genes in class IV exhibited a considerable level of expression in root, and the majority of class III chitinase (14/19) shows a low expression level in unstressed seedlings (Figure 7). Most of the chitinases with very high expression level under cold stress belong to class III (Figure 8). *EVM0022783*, *EVM0020238*, and *EVM0008380*, the three chitinases clustered into a small clade in phylogenetic tree, were all induced by cold stress (Figure 2 and Figure 8), while *EVM0034210* and *EVM0012833*, two chitinases similar in sequence, were down-regulated under cold stress (Figure 2 and Figure 8). These findings might suggest that the regulatory sequences involved in stress response in the promoter region did not diverge much along with the evolution of these chitinases genes after duplication.

*A. nanus* exhibits an extremely high level of drought and low temperature tolerance, thus, it is not surprisingly to find that the MYC/ICE1 binding site, a cis-acting element involved in the response to dehydration and low temperature, is present in promoters of 28 (87.5% of total) chitinases (Figure 6). Moreover, 9 of these 28 chitinases carry more than 9 copies of MYC/ICE1 binding sites. The C-repeat (CRT) binding factors (CBFs) play crucial roles in plant cold response and acclimation [66], and Inducer of CBF expression 1 (ICE1), a MYC-like basic-helix-loop-helix transcription factor, regulates the expression of CBF by binding to the MYC/ICE1 binding site in CBF promoters [67]. Our results indicate that most of the chitinases were involved in the low-temperature signaling pathway via regulation by ICE1. ICE1 is a positive regulator in the low temperature signal transduction pathway. Under low temperature stress, the activated ICE1 directly binds to the ICE1 binding site and promotes the expression of downstream genes such as CBFs [66]. This study showed that some of the MYC/ICE1 binding site was distributed in promoters of chitinase gene in *A. nanus*, and might mediate the induction of the chitinase gene under low temperature stress. Of the eight chitinase genes whose promoters harbored more than nine copies of MYC/ICE1 binding sites, six were up-regulated under low-temperature stress (Figure 6 and Figure S2). Moreover, among the four chitinases whose promoters did not contain an MYC/ICE1 binding site, three, i.e., *EVM0009532*, *EVM0034210*, and *EVM0026818,* were down-regulated under low-temperature stress (Figure 6 and Figure S2). These data supported the essential role of the MYC/ICE1 binding site in the induction of chitinase genes in *A. nanus*. In sum, the chitinases induced by low temperature and carrying multiple MYC/ICE1 binding sites in their promoters, such as *EVM0022783*, *EVM0020238*, and *EVM0003645*, are likely to play important roles in the low temperature and drought tolerance of *A. nanus*. These low temperature and osmotic stress-induced chitinases should be ideal candidate genes used for genetic engineering for improving abiotic stress tolerance of crops and other economic plants.

Almost all low temperature-induced chitinase genes were also up-regulated under osmotic stress, indicating that these chitinases were involved in the response to both low temperature and osmotic stress probably through the signaling pathway mediated by ICE1 [67]. As is a rare evergreen broad-leaved shrub distributed in arid region in Central Asia, one of the most critical environmental factors affecting the survival of the plant is the freezing stress up to −15 °C at night in winter. Therefore, that almost all low temperature-induced chitinases were also induced by osmotic stress is beneficial for *A. nanus* to resist the severe dehydration stress caused by freezing stress. 

Another one of the most frequently occurring cis-acting elements in promoters of chitinase family in *A. nanus* is TGAC-containing W-box, which is present in promoters of all *A. nanus* chitinases (Figure 6). Given that TGAC-containing W-box has been shown to mediate the fungal elicitor-induced gene expression by interacting with WRKY1 in parsley [68], all *A. nanus* chitinases might contribute to the defense to fungal pathogen via the regulation by WRKY transcription factors. There are multiple copies (2-12) of the TGAC-containing W-box in the promoter region of chitinase, which is consistent with the commonly accepted opinions that chitinase plays an important role in antipathogenic fungi.

ARR1-binding element is also present in the promoter of all chitinase genes in *A. nanus,* with a copy number ranging from 1 to 20. Arabidopsis response regulator protein (ARR1, AT3G16857) functions as a response regulator involved in His-to-Asp phosphorelay signal transduction system and plays a vital role in the cytokinin signaling pathway [69]. Cytokinins have been implicated in various developmental and physiological processes of plants [70], and our results indicates that chitinases might also be regulated by the cytokinin signaling pathway, suggesting a cross talk between abiotic stress and cytokinin signaling in *A. nanus*.

In brief, the expression profiling of the chitinases under low temperature and osmotic stress will be helpful to understanding the biological function of the individual chitinase. In the next step, we will further investigate the biological functions of stress-induced chitinases in stress adaptation in *A. nanus* by, for example, identifying the interacting proteins of these chitinases via pull-down assay. 

## 5. Conclusions

Here, a total of 32 chitinase genes were identified from the genome sequence of *A. nanus*, and out of these chitinases, 24 were classified into the GH18 subfamily, and eight into the GH19 subfamily. There is a single Class I, 4 Class II, 19 Class III, 3 Class IV, and 5 Class V chitinases in *A. nanus*. We noticed that the number of Class III of *A. nanus* was significantly higher than that of other plant species like *A. thaliana*, suggesting that class III chitinase genes may be amplified in *A. nanus*. Class III chitinase genes also contains less introns, indicating their involvement in stress response in *A. nanus*. Expression profiling of chitinases under low temperature and osmotic stress, as well as the prediction of the cis-acting elements in promoter region of chitinase, help to identify chitinases that might play key roles in the abiotic stress responses in *A. nanus*. These results could provide important data for understanding the diversifying functions of plant chitinases and will be helpful for identification of the novel chitinase genes that can be used for both disease control and enhancement of abiotic stress tolerance. 

## Figures and Tables

**Table 1:** Characterization of predicted chitinases in *A. nanus*.

Sequence ID	Genome Location	Domains	Class	Amino Acid Length	Signal Peptide	Number of Introns	Chitin Binding Region	Predicted pI	Predicted Mw
EVM0015141	ch06:74872028--74873992	GH19	I	344	1-24	4	50--69	8.42	36,164.62
EVM0000245	ch01:82754230--82758136	GH19	II	328	1-24	4	NO	5.82	35,570.39
EVM0010035	ch03:67952710--67953891	GH19	II	270	1-17	2	NO	5.81	29,087.94
EVM0003834	ch03:89647173--89650518	GH19	II	326	1-21	4	NO	7.48	34,959.76
EVM0030372	ch06:24817881--24818291	GH19	II	139	1-29	0	NO	8.58	15,484.92
EVM0003649	ch04:80079800--80081378	GH19	IV	279	1-26	3	31--50	4.70	29,787.24
EVM0034576	ch08:83000063--83004274	GH19	IV	291	NO	3	40--64	4.22	30,666.83
EVM0009532	ch08:83008232--83009062	GH19	IV	245	NO	1	NO	4.88	26,440.78
EVM0037111	ch01:19478187--19479445	GH18	III	309	1-27	2	NO	6.43	32,622.71
EVM0004538	ch01:19470407--19471892	GH18	III	271	1-23	3	NO	5.83	28,923.78
EVM0017249	ch01:24538090--24543941	GH18	III	119	NO	2	NO	5.10	12,987.12
EVM0024770	ch03:2089454--2095600	GH18	III	441	1-20	9	NO	8.59	49,055.54
EVM0011210	ch03:2653696--2655594	GH18	III	296	1-28	2	NO	4.87	31,084.80
EVM0024636	ch03:15250701--15252121	GH18	III	305	1-29	2	NO	5.27	33,518.83
EVM0009398	ch06:24575731--24576843	GH18	III	308	1-25	2	NO	6.80	31,905.08
EVM0008380	ch07:59025620--59027551	GH18	III	404	1-27	1	NO	5.46	43,543.43
EVM0020238	ch07:57782685--57783731	GH18	III	356	1-30	0	NO	4.56	38,587.45
EVM0015492	ch08:57768990--57769886	GH18	III	306	1-23	0	NO	8.92	32,347.84
EVM0014498	ch09:11798331--11799544	GH18	III	307	1-28	2	NO	8.97	32,399.49
EVM0022783	ch09:80432948--80446622	GH18	III	346	1-27	3	NO	5.17	37,050.17
EVM0034210	ch09:80420661--80421764	GH18	III	305	1-24	2	NO	5.04	31,361.14
EVM0028584	ch09:80454721--80456132	GH18	III	308	1-22	2	NO	8.98	32,352.94
EVM0012833	ch08:57760416--57761582	GH18	III	307	1-25	2	NO	4.44	31,602.09
EVM0026818	ch05:6506791--6507856	GH18	III	315	1-29	1	NO	5.56	33,909.98
EVM0003645	ch08:63789032--63793200	GH18	III	804	1-29	8	NO	8.35	88,243.97
EVM0017185	ch08:63796631--63800128	GH18	III	751	1-31	7	NO	8.29	82,885.73
EVM0036771	ch04:6503824--6505708	GH18	III	269	NO	3	NO	5.77	30,665.56
EVM0012914	ch02:67985626--67991461	GH18	V	390	1-20	2	NO	8.63	41,046.00
EVM0003584	ch02:67952710--67953891	GH18	V	341	1-24	1	NO	6.81	36,866.35
EVM0013536	ch04:79575721--79578939	GH18	V	379	1-21	2	NO	9.12	40,742.48
EVM0019404	ch05:65508611--65510368	GH18	V	393	1-21	3	NO	7.78	43,121.80
EVM0018581	ch01:67959345--67975423	GH18	V	1027	1-22	12	NO	7.25	114,928.43

pI: isoelectric point; Mw: molecular weight.

**Table 2:** Number of each chitinase class in *A. nanus*, *A. thaliana*, *B. rapa*, *P. trichocarpa*, *E. grandis, G. raimondii*, *B. juncea*, *C. sativa*, and *H. brasiliensis*.

	*A. nanus*	*A. thaliana*	*B. rapa*	*P. trichocarpa*	*E. grandis*	*G. raimondii*	*H. brasiliensis*	*B. juncea*	*C. sativa*
Class I	1	1	5	11	1	5	7	10	4
Class II	4	2	5	3	6	5	1	6	21
Class III	19	1	2	13	10	10	16	2	3
Class IV	3	9	17	5	14	7	5	25	26
Class V	5	9	4	5	26	19	10	4	25
Total	32	24	33	37	67	47*	39	47	79

* One chitinase is not categorized into any class in *G. raimondii* [31].

## Supplementary Materials

The following are available online at https://www.mdpi.com/2073-4425/10/6/472/s1, Figure S1: Expression patterns of *A. nanus* chitinases in leaves, stems, and roots, Figure S2: Expression profiles of *A. nanus* chitinases under low temperature, Figure S3: Expression profiles of *A. nanus* chitinases under osmotic stress, Table S1: The primers used for qRT-PCR analysis of *A. nanus* chitinases.
