# Supplementary material for: Genome-Wide Identification and Expression Analyses of the Chitinases under Cold and Osmotic Stress in Ammopiptanthus nanus
Source: Genes (Basel). 2019 Jun 21;10(6):472. doi: 10.3390/genes10060472 (PMC6627877; doi:10.3390/genes10060472)
Supplement: Supplementary file 1 [file genes-10-00472-s001.zip › Supplementary material.docx]

Supplementary material

Table S1. The primers used for qRT-PCR analysis of A. nanus chitinases

| **Gene** | **Forward primer** | **Reverse primer** |
| --- | --- | --- |
| *EVM0014498* | tggtgatgctgtattagatgggg | gcatgttcattcagcctcttagc |
| *EVM0010035* | ttgggagttgaagcttgggg | gggcatgcattgtcatctttgt |
| *EVM0026818* | gatcctgatacctttgctgagtg | atagaagcaaaagtgatgacccc |
| *EVM0008380* | ggatggcattgatatagcagacg | tcaattgtggactggttaatggc |
| *EVM0012833* | ccattgggtgatgcagttttgg | aaaccgttgagtgccctgac |
| *EVM0015141* | tgcggtaccacagatccttact | gggtgtgggtttgggagttg |
| *EVM0012914* | tattctggtgttggccctgc | ggagggtgcattggaagacg |
| *EVM0034576* | ttgtgggagcagtgaggagt | tcgttggttgaagttgaggca |
| *EVM0003649* | cgggtattgtgggaacggtg | gttgggtggactttggctcc |
| *EVM0000245* | gctcatgaggccttcgttgg | tcaacatcaccttgcccaca |
| *EVM0015492* | ctcgccttcacgcccattt | agataacttgcaaggctgtccc |
| *EVM0019404* | taccagagttcatcggcggg | acaaagtggagttgctgcca |
| *EVM0022783* | accacaaatttactacctcggcc | tcttgtgccagctgaacttga |
| *EVM0037111* | ggggatgctgcattagatgga | cgttcggtgagtctcttagca |
| *EVM0034210* | gtgatgctgtgttggatggc | ctgaacccatcgagtgcctt |
| *EVM0013536* | acggatcaaatgggacaaag | cccgaatacgaatacgctgt |
| *EVM0011210* | tggaataacttcttgggaggaca | ggtttgttcctccttctatgtca |
| *EVM0004538* | acaggctcaaatcagtactggg | cctgcccacgaattcttcca |
| *EVM0024770* | tgaaatggcaaagagggtcaact | tgagatccatcctctgtcagca |
| *EVM0024636* | acttcaatgaatcttgcggctg | ttcgatggagcaatggaggc |
| *EVM0017249* | gcatggaagcaatggacatca | cctcacacacagtcagggct |
| *EVM0003834* | ccaagcgggttcctggattt | actcattatcagatccttggcca |
| *EVM0028584* | gcaagtcatcttcgaggcca | ccctccttcctaccctgctata |
| *EVM0009398* | cagtcatcatctcgtccacttgg | ccccagtaatggtttgatcctcc |
| *EVM0030372* | ttcttaggacacgttggcagc | agagcaccgcgtccatagtaa |
| *EVM0009532* | cttggtgttgctcctggtgat | accctgaataaacaagagctgac |
| *EVM0020238* | gaagtgtttccttggatggca | tgtggactcgttaatggcct |
| *EVM0003645* | ttctagcaatgggtgcactg | ttagcatctgccgaggaagt |
| *EVM0017185* | acttcctcagcggatgctaa | atgggcgtgaagaagagttg |
| *EVM0036771* | gagaaggatgttcccaccaa | gttctcttgggggctaaagg |
| *EVM0018581* | ggttttgatgatgccaaggt | gctgctgcactagaaagcac |
| 18S rRNA | ctgacatgcgccgtaggaacg | ccctgcttatgccgagtctttt |

| A |  |  |  |
| --- | --- | --- | --- |
| 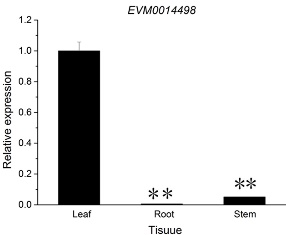 | 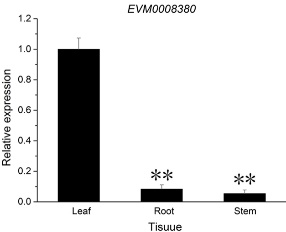 | 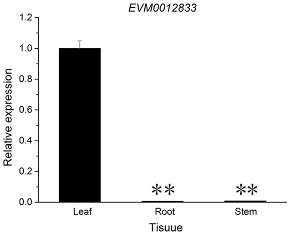 | 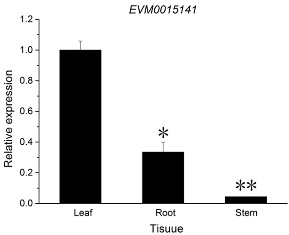 |
| 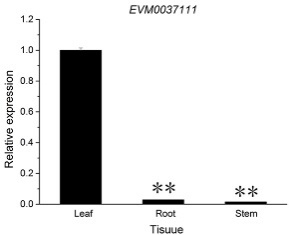 | 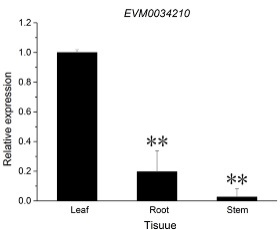 | 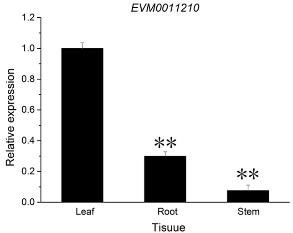 | 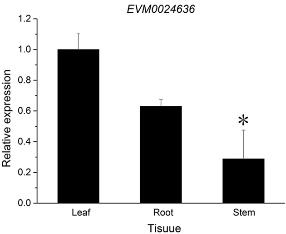 |
| 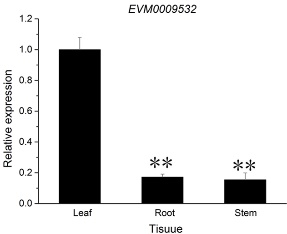 | 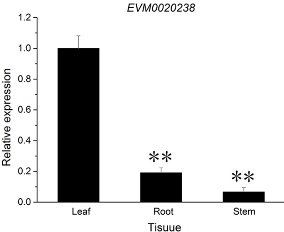 |  |  |
| B |  |  |  |
| 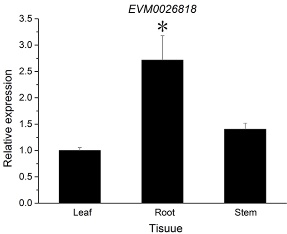 | 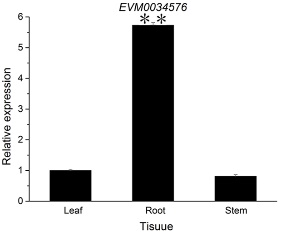 | 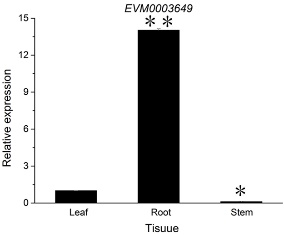 | 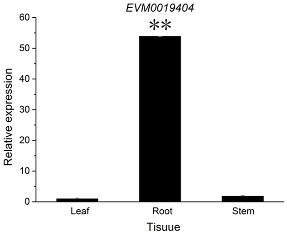 |
| 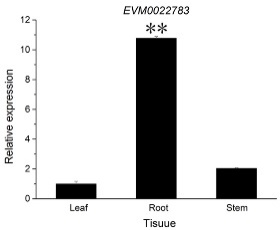 | 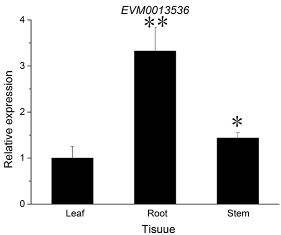 | 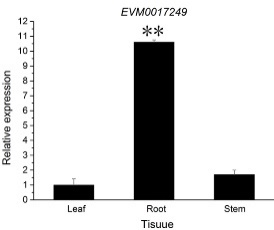 | 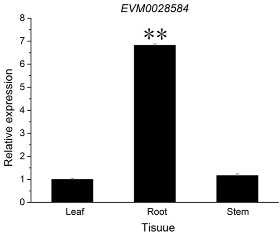 |
| 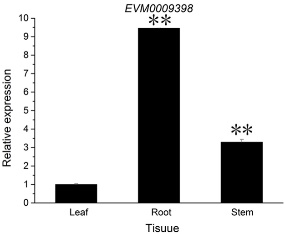 | 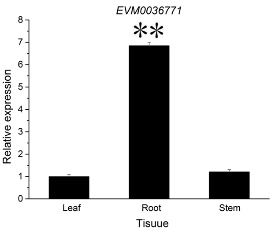 | 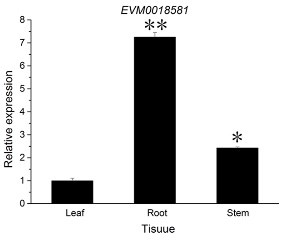 |  |
| C |  |  |  |
| 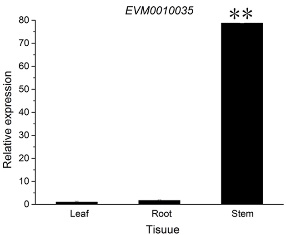 | 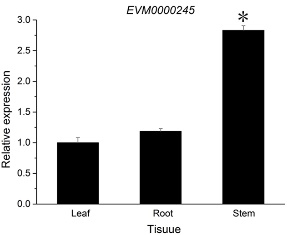 | 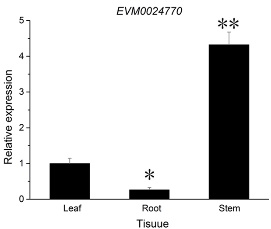 |  |
| D |  |  |  |
| 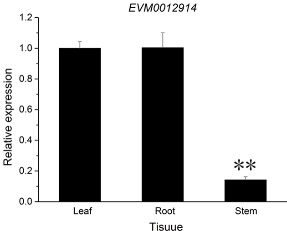 | 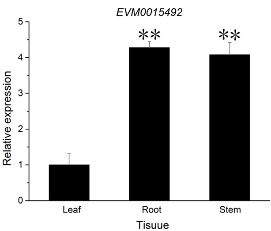 | 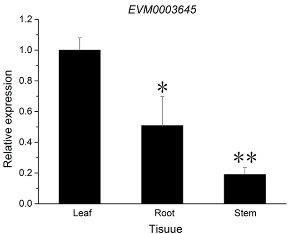 | 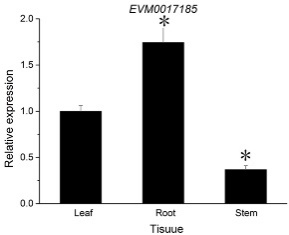 |
| 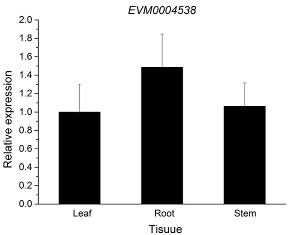 | 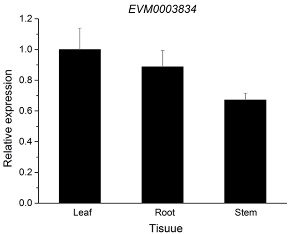 | 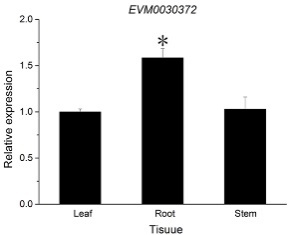 |  |

Figure S1. Expression patterns of *A. nanus* chitinases in leaves, stems, and roots. The chitinases expressed dominantly in leaves (A), roots (B), stem (C), or expressed considerably in at least two tissues (D). *A. nanus* elF1 was used as the internal control. *, p < 0.05; **, p < 0.01, compared with the control group. Error bar represent standard error for three replicates.

| A |  |  |  |
| --- | --- | --- | --- |
| 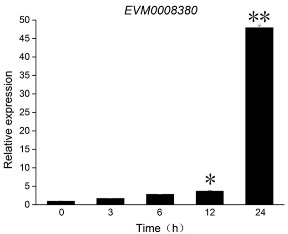 | 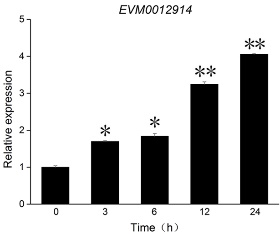 | 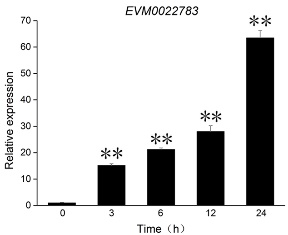 | 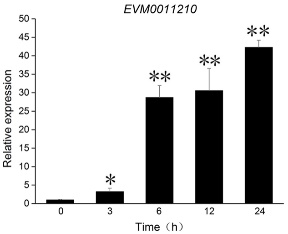 |
| 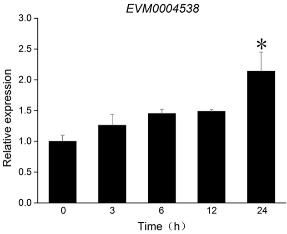 | 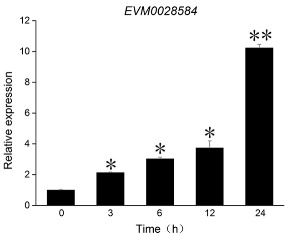 | 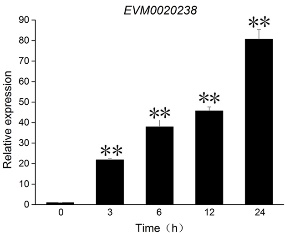 | 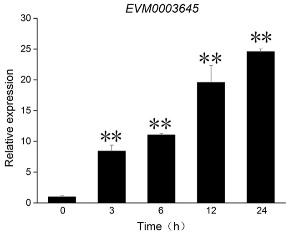 |
| 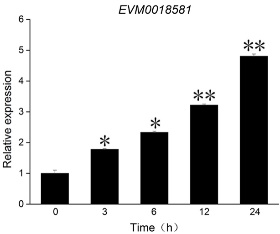 | 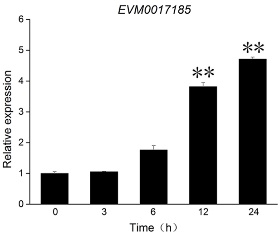 |  |  |
| B |  |  |  |
| 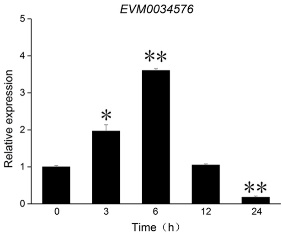 | 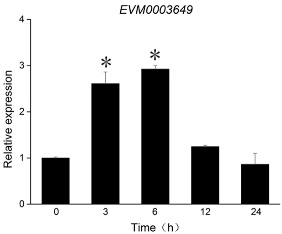 | 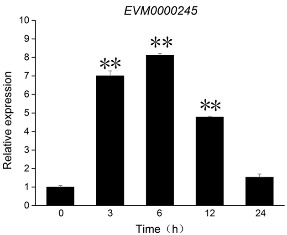 | 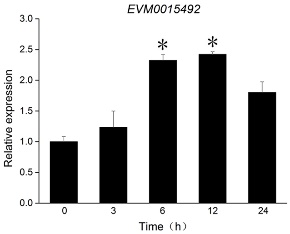 |
| 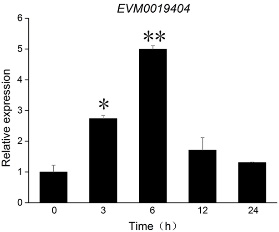 | 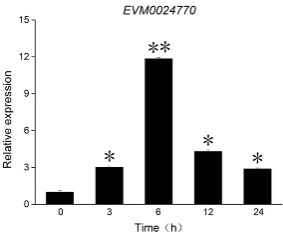 | 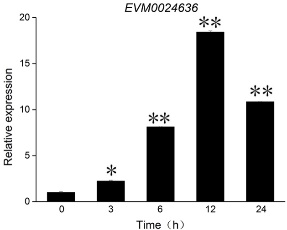 | 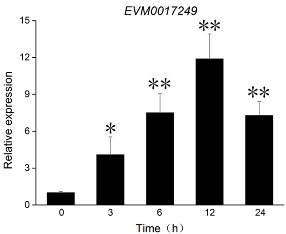 |
| 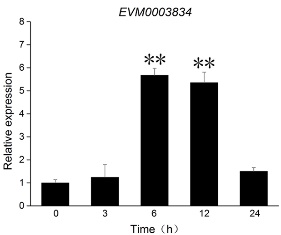 | 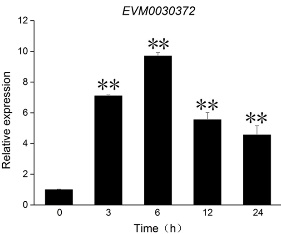 |  |  |
| C |  |  |  |
| 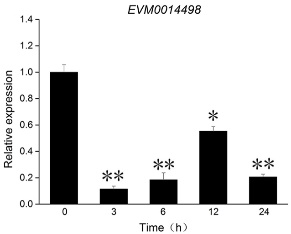 | 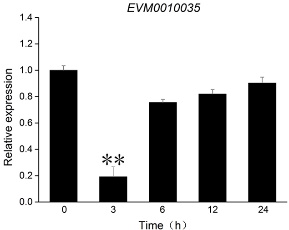 | 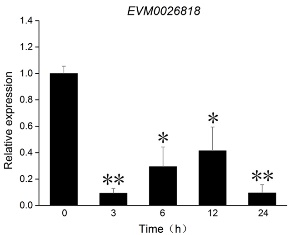 | 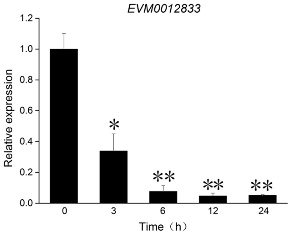 |
| 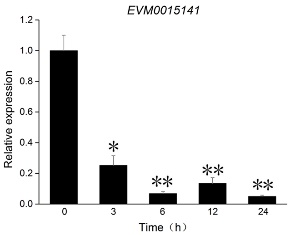 | 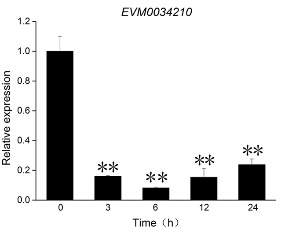 | 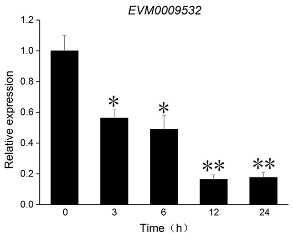 | 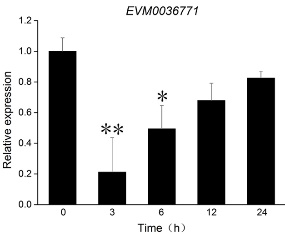 |
| D |  |  |  |
| 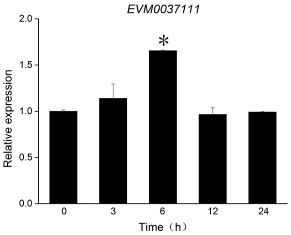 | 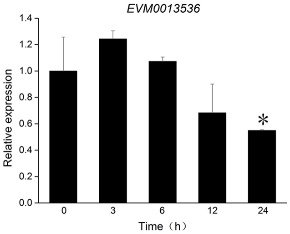 | 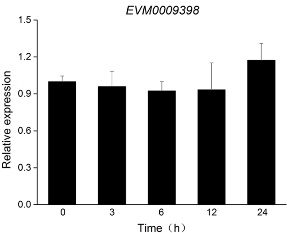 |  |

**Figure S2**. Expression profiles of *A. nanus* chitinases under low temperature. The expression pattern shows a linear upward curve (A), or inverted U-shaped curve (B). The expression levels were down-regulated during at least one time-point (C), or did not show significant change (up or down-regulated by >2-fold) under low temperature stress (D). *A. nanus* elF1 was used as the internal control. *, p < 0.05; **, p < 0.01, compared with the control group. Error bar represent standard error for three replicates.

| A |  |  |  |
| --- | --- | --- | --- |
| 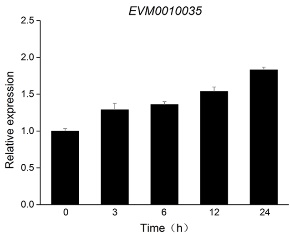 | 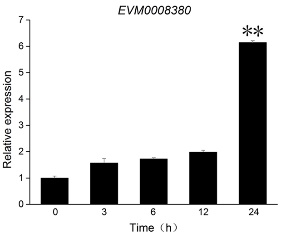 | 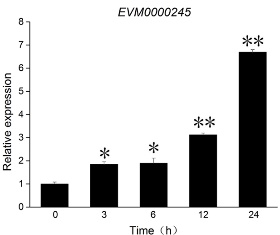 | 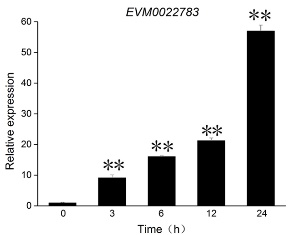 |
| 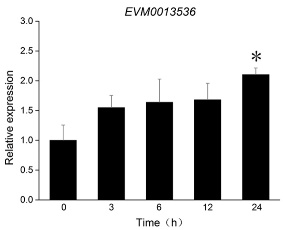 | 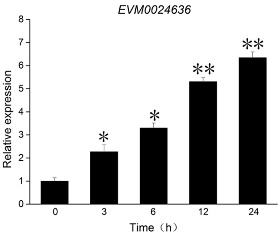 | 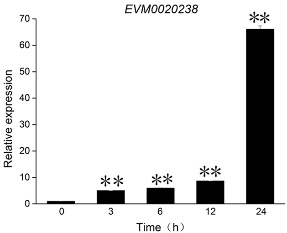 |  |
| B |  |  |  |
| 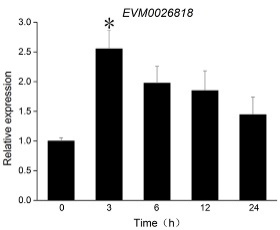 | 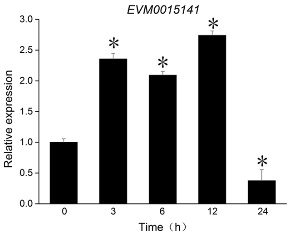 | 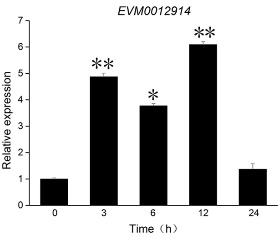 | 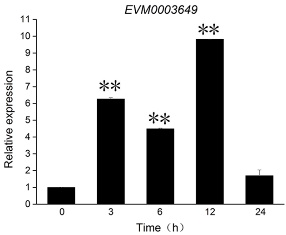 |
| 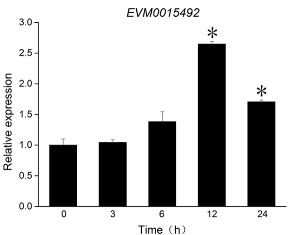 | 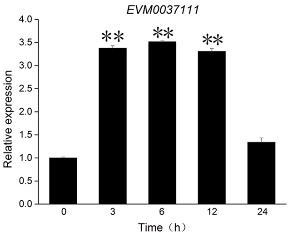 | 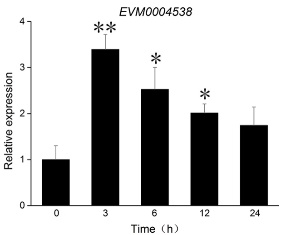 | 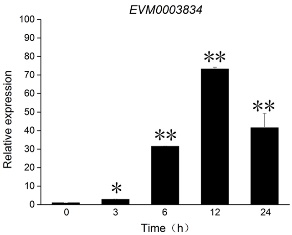 |
| 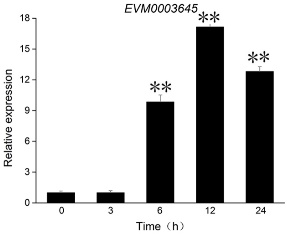 | 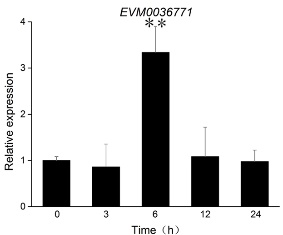 | 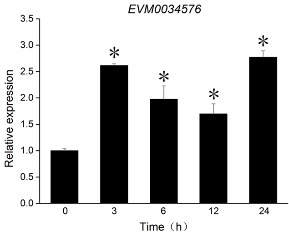 | 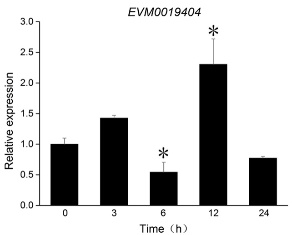 |
| 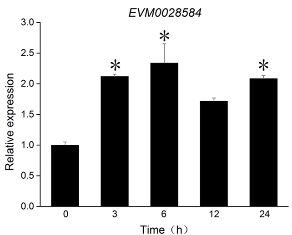 | 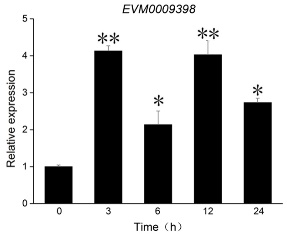 | 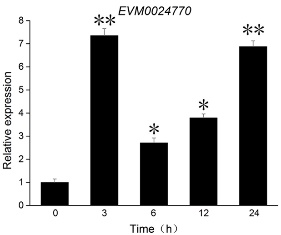 | 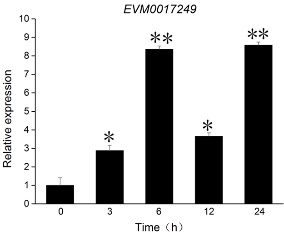 |
| 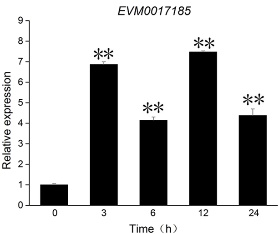 | 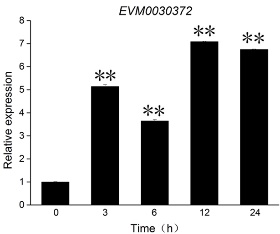 | 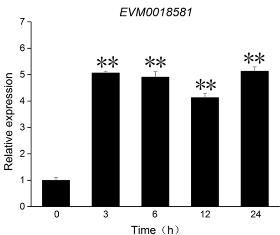 |  |
| C |  |  |  |
| 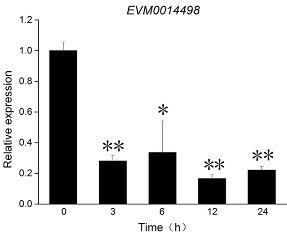 | 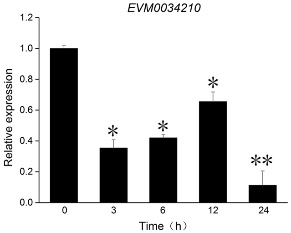 | 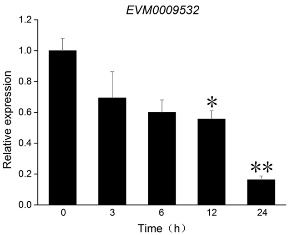 | 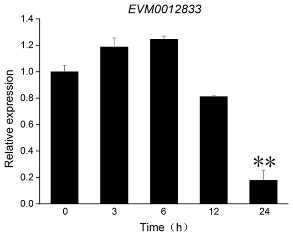 |
| D |  |  |  |
| 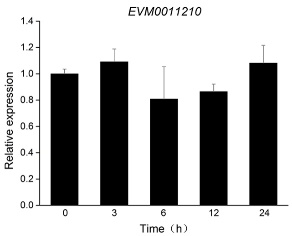 |  |  |  |

**Figure S3**. Expression profiles of *A. nanus* chitinases under osmotic stress. The expression pattern shows a linear upward curve (A). The expression levels were up-regulated by > 2-fold during at least one time-point under osmotic stress, but cannot be classified into the first group (B). The expression levels were down-regulated (C), or the expression levels did not show significant change (up or down-regulated by >2-fold) under osmotic stress (D). *A. nanus* elF1 was used as the internal control. *, p < 0.05; **, p < 0.01 compared with the control group. Error bar represent standard error for three replicates.
